# The Niagara University—The Medical Department

**Published:** 1883-09

**Authors:** 


### The Niagara University—The Medical Department.

Acting under a charter granted by the regents of the University of the State of New York, the trustees of Niagara University have decided to open, at an early day, the department of medicine, in connection with the hospital of the Sisters of Charity, in this city. This movement has been under advisement for several months, and has been brought to a favorable termination through the earnest efforts of the trustees of the above-named institution, who have been actuated by a desire to give a practical response to the general demand, so universally prevailing in the medical profession, for increased opportunities and facilities for the higher education of medical men. The new medical college opens, therefore, under the most favorable auspices, by being connected with the large hospital of the Sisters of Charity, which will furnish ample clinical material for its classes, commodious lecture-rooms, chemical and physiological laboratories, and the most approved facilities for the great work it undertakes to do for the profession. This institution opens its course with requirements which come up to the principles which the Journal has heretofore advocated. It requires its matriculants to furnish evidence of a certain and reasonable standard of literary attainments before entering upon the study of medicine. It lays down a graded curriculum of study patterned after the best medical colleges in this country; it provides for a thorough examination of the student in passing to the higher classes, and finally it submits its candidates for graduation to a Board of Medical Examiners, outside of the faculty, upon whose examination the application for the degree is to be accepted or rejected.

It will be seen that these principles are in the interests of higher medical education. The college, therefore, is not brought into rivalry with other medical schools, simply for the reason that in this vicinity, on account of its high standard of requirements, there is nothing with which it is brought into competition. Its standard of admission, its graded curriculum and its examination for degrees before an independent board of examiners, place the institution upon a basis which must command the support and approval of the profession in whose interests it will labor.

The following gentlemen compose its faculty:

John Cronyn, M. D., Professor of the Theory and Practice of Medicine.

C. C. F. Gay, M. D., Professor of Operative and Clinical Surgery.

William S. Tremaine, M. D., U. S. A., Professor of the Principles and Practice of Surgery and Clinical Surgery.

Thomas Lothrop, M. D., Professor of Obstetrics.

Augustus R. Davidson, M. D., Professor of Medical Chemistry, Pharmacy and Toxicology.

George E Fell, M. D., Professor of Physiology and Microscopy.

William H. Heath, M. D., Professor of Anatomy.

Clayton M. Daniels, M. D., Professor of Clinical Surgery, and Adjunct Professor of Surgery.

Henry D. Ingraham, M D., Professor of Gynecology and Diseases of Children.

Alvin A. Hubbell, M. D., Professor of Ophthalmology, Otology and Laryngology.

Charles G. Stockton, M. D., Professor of Materia Medica and Therapeutics.

Hon. Joseph M. Congdon, Professor of Medical Jurisprudence.

These gentlemen are well and favorably known to the profession and to the public, and their reputation gives assurance that the principles on which the new college is based will be faithfully carried out. The large and prosperous hospital which furnishes accommodations for clinics, lectures, laboratory, etc., gives character and stability to the movement which commends it to our confidence and is a sure augury of success.

We shall refer to this important step in the interests of the higher education of the profession at another time, giving further details of its progress. The new college opens its course October 10, the amphitheatre at the hospital being used for the lecture room, while commodious laboratories have been fitted up for chemical and physiological purposes.

